# Educational Inequalities in Exit from Paid Employment among Dutch Workers: The Influence of Health, Lifestyle and Work

**DOI:** 10.1371/journal.pone.0134867

**Published:** 2015-08-07

**Authors:** Suzan J. W. Robroek, Anne Rongen, Coos H. Arts, Ferdy W. H. Otten, Alex Burdorf, Merel Schuring

**Affiliations:** 1 Department of Public Health, Erasmus MC, University Medical Center Rotterdam, Rotterdam, the Netherlands; 2 Statistics Netherlands, Heerlen, the Netherlands; Hunter College, UNITED STATES

## Abstract

**Background:**

Individuals with lower socioeconomic status are at increased risk of involuntary exit from paid employment. To give sound advice for primary prevention in the workforce, insight is needed into the role of mediating factors between socioeconomic status and labour force participation. Therefore, it is aimed to investigate the influence of health status, lifestyle-related factors and work characteristics on educational differences in exit from paid employment.

**Methods:**

14,708 Dutch employees participated in a ten-year follow-up study during 1999–2008. At baseline, education, self-perceived health, lifestyle (smoking, alcohol, sports, BMI) and psychosocial (demands, control, rewards) and physical work characteristics were measured by questionnaire. Employment status was ascertained monthly based on tax records. The relation between education, health, lifestyle, work-characteristics and exit from paid employment through disability benefits, unemployment, early retirement and economic inactivity was investigated by competing risks regression analyses. The mediating effects of these factors on educational differences in exit from paid employment were tested using a stepwise approach.

**Results:**

Lower educated workers were more likely to exit paid employment through disability benefits (SHR:1.84), unemployment (SHR:1.74), and economic inactivity (SHR:1.53) but not due to early retirement (SHR:0.92). Poor or moderate health, an unhealthy lifestyle, and unfavourable work characteristics were associated with disability benefits and unemployment, and an unhealthy lifestyle with economic inactivity. Educational differences in disability benefits were explained for 40% by health, 31% by lifestyle, and 12% by work characteristics. For economic inactivity and unemployment, up to 14% and 21% of the educational differences could be explained, particularly by lifestyle-related factors.

**Conclusions:**

There are educational differences in exit from paid employment, which are partly mediated by health, lifestyle and work characteristics, particularly for disability benefits. Health promotion and improving working conditions seem important measures to maintain a productive workforce, particularly among workers with a low education.

## Introduction

Individuals with lower socioeconomic status are at increased risk of involuntary exit from paid employment [1–6]. Premature exit from paid employment might deteriorate the health of the former workers [7], contributing to widening socioeconomic health inequalities. There are also large socioeconomic inequalities in determinants of labour force participation, i.e. health status, lifestyle, and psychosocial and physical work characteristics [8]. However, the contribution of these factors to socioeconomic differences in labour force participation is largely unknown. To give sound advice for primary prevention in the workforce, firstly insight is needed into the role of mediating factors between socioeconomic status and labour force participation.

A recent meta-analysis showed that poor health is an important barrier for maintaining paid employment [9]. Particularly workers with a lower socioeconomic status, defined by low educational level, occupational class or income, are at increased risk for health-based selection out of employment [2,4]. Important modifiable determinants of sustainable employability include lifestyle-related factors and work characteristics. It is well-known that individuals with a lower socioeconomic status are more likely to have an unhealthy lifestyle and a poorer quality of work, i.e. unfavourable psychosocial and physical work demands [8,10], which are risk factors for premature exit from paid employment, particularly a lack of physical activity and low job control [3,11,12]. However, the relative importance of health problems, lifestyle-related factors and work characteristics differs between involuntary (i.e. disability benefits, unemployment) and voluntary (i.e. early retirement, economic inactivity) exit routes from paid employment [3,4]. Disability benefits is typically a health-driven exit pathway. Individuals who are incapable to work due to health problems may be eligible to receive a disability benefit. In general, disability benefits are higher than benefits for unemployment or early retirement. Individuals with health problems, but who do not receive a disability benefit, might leave the workforce via the other exit routes more or less voluntarily. Older persons having health problems might retire early, while others might become economically inactive. Most studies focus on a single exit route or on multiple routes as completely independent events. However, this might result in biased estimates, asking for novel models taking into account competing risks between exit routes [13].

Studies investigating the contribution of health, lifestyle-related factors and work characteristics in socioeconomic differences in labour force participation are mainly restricted to sickness absence [14–17] and exit from paid employment through disability benefits [18,19]. These studies report that preventing ill health might help to reduce socioeconomic inequalities in disability benefits [19]. However, there is a lack of insight to what extent these factors explain the relation between socioeconomic status and the competing routes of exit from paid employment, such as disability benefits and unemployment.

The objective of this study is to get insight into effects of health, lifestyle-related factors, and work characteristics on the relation between educational level and exit from paid employment. Therefore, we investigate whether educational differences in exit from paid employment are mediated by health status, lifestyle-related factors, and work characteristics. We hypothesize that the contributions of these factors differ per pathway of exit from paid employment, and that particularly health, and to a lesser extent lifestyle-related factors and work characteristics, contribute to educational inequalities in exit from paid employment.

## Materials and Methods

### Design and study population

The study population was based on an annual national survey “Permanent Survey on Living Conditions” (POLS) carried out among a random sample of the non-institutionalised individuals in the Netherlands by Statistics Netherlands in the period 1999–2002 [20]. The yearly response to the POLS survey is approximately 60–65%. In total, 39,220 persons responded to the survey between 1999 and 2002. The POLS data were enriched by Statistics Netherlands with information on the main income components, i.e. social benefits, pensions and gross wages, derived from Dutch tax registers and stored in a social statistical database (SSB). Subsequently, the POLS-data were longitudinally matched with the SSB- for each subsequent month during a ten year follow up period (1999–2008). For the purpose of this study, 14,708 individuals (93,960 person-years) aged between 18–64 years who were in paid employment for at least 12 hours per week in the Netherlands at the time of the health survey and filled out questions on health, lifestyle and work, were selected. In the Netherlands there are no possibilities to officially retire before the age of 50 and the statutory retirement age was 65 years. Therefore, the study population in the analyses on early retirement was restricted to individuals aged 50–64 years (n = 2,922). A passive informed consent procedure was used in which participants were informed about the linkage of their questionnaire information with register data.

The used data are stored in the Social Statistical Database of Statistics Netherlands, The Hague, the Netherlands. Interested researchers may submit requests for remote access data-analysis to cvb@cbs.nl.

#### Exit from paid employment

Information on the income components was derived from the Dutch tax register as provided by Statistics Netherlands. Employment status was divided into five mutually exclusive categories: employment, disability benefits, unemployment, early retirement, and economically inactive. Employed individuals had their main source of income through paid employment.

In the Netherlands, individuals who are partially or fully incapable of working after two years of illness become eligible to receive a disability benefit. During the first two years of sickness absence the employer has to pay the salary of the sick employee and during this period the employee is still classified as being employed. Thereafter, the degree of the disability is determined by the loss of earnings due to illness relative to the earning before. Only when there is a reduction of income greater than 35%, disability benefits will be granted (www.government.nl). In this study, exit from paid employment through disability benefits is defined as receiving benefits for at least 50% of their personal income. Unemployed persons received unemployment benefits or social security benefits. In the Netherlands individuals receive unemployment benefits in case of loss of paid employment, with a maximum of 38 months. After this period the corresponding household may receive a social security benefit in case the disposable (household) income is below the legislative threshold (www.government.nl). Early retired individuals received a (pre-)pension as their main income before they reached the age of 65 years. Economically inactive individuals had no personal income and did not receive any benefits. These individuals may have left paid employment voluntarily or may belong to a household whose disposable income is above the critical threshold for social security benefits.

### Socio-demographics

Individual characteristics included age, sex, educational level, and marital status. The highest level of education was coded according to the 1997 International Standard Classification of Education (ISCED-97) and categorized into low (pre-primary, primary and lower secondary), intermediate (upper secondary) and high (post-secondary) education. Marital status was used to categorize individuals into those who were living with a spouse or partner in the same household and those living alone.

### Health and lifestyle-related factors

Self-perceived health status was assessed by a single question asking individuals to rate their overall health on a five-point scale [21]. The answer categories were dichotomized into ‘very poor, poor, or moderate’ and ‘good or very good’. Body mass index (BMI) was calculated based on self-reported height in meters and weight in kilograms and categorized into underweight (<18.5kg/m^2^), normal weight (18.5-<25kg/m^2^), overweight (≥25–<30kg/m^2^), and obese (≥30kg/m^2^) [22]. Participation in sports was measured by asking how many hours on how many days per week the individuals engage in sports. Those individuals who reported to participate less than one hour per week were considered to have a lack of sports participation, most closely corresponding with the updated recommendation for vigorous physical activity, i.e. at least three times a week 20 minutes [6,23]. Individuals answering the single-item question “Do you ever smoke?" with ‘yes’ were considered smokers. Alcohol intake was assessed by asking how many glasses of alcoholic beverages one drinks per week. Heavy alcohol intake was defined as drinking >14 glasses (women) and >21 glasses (men), using cut-offs of Dutch guidelines [24].

### Work characteristics

Psychosocial workload was measured by work demands, job control, and rewards. Work demands were measured by using two items (Cronbach’s alpha:0.76) concerning a) working at a high pace, and b) working under time pressure. A three-point scale was used with ratings (1) ‘regularly’, (2) ‘sometimes’, and (3) ‘no’. A sum score was calculated and workers with a sum score in the lowest quartile were regarded as having high work demands. Job control was assessed using five items (Cronbach’s alpha:0.66) concerning a) workers’ influence on their work regarding the work pace, b) execution of their work, c) order of tasks, d) interruption when needed, and e) finding solutions. The three answer categories were (1) ‘regularly’, (2)‘sometimes’, and (3) ‘no’. The answers were re-coded, in such a way that in the sum score the highest quartile was regarded as having low job control. Rewards were assessed by a single item asking to whether the worker was satisfied with the salary. Workers answering the question with ‘no’ were regarded as having low rewards.

Physical work demands were measured using three items (Cronbach’s alpha:0.84) concerning a) physically demanding work in general, b) activities such as heavy lifting, pulling or pushing, or use of heavy machinery, and c) work that makes the worker sweat or out of breath. A three-point scale was used with ratings (1) ‘regularly’, (2) ‘sometimes’, and (3) ‘no’. A sum score was calculated and workers with a sum score in the lowest quartile were regarded as having high physical work demands.

### Statistical analysis

Descriptive statistics were used to present exit from paid employment stratified by educational level. The rate of exit is presented as number of exit events per 1000 person-years.

Associations between education and health, lifestyle and work characteristics at baseline were analysed using logistic regression analyses, adjusting for sex, age, and marital status. Thereafter, the effects of education, poor health, unhealthy lifestyle and unfavourable work characteristics on exit from paid employment during the 10 year follow-up period were analysed using competing risks regression analyses based on Fine and Gray’s proportional subhazards model [13], adjusting for sex, age, and marital status. The likelihood of the occurrence of an event was estimated, taking into account the likelihood that another event may prevent the occurrence of the event of interest. Each pathway of exit from paid employment (e.g. disability benefits) was subsequently included as the event of interest, whereas the other pathways out of the labour force were included in the analyses as competing events. An individual was censored at the moment the individual reached the retirement age, died, left the country, started with education, or at the end of the follow-up period. A subdistribution hazard ratio (SHR) greater than one indicates an increased likelihood of exit from paid employment.

**Fig 1:**
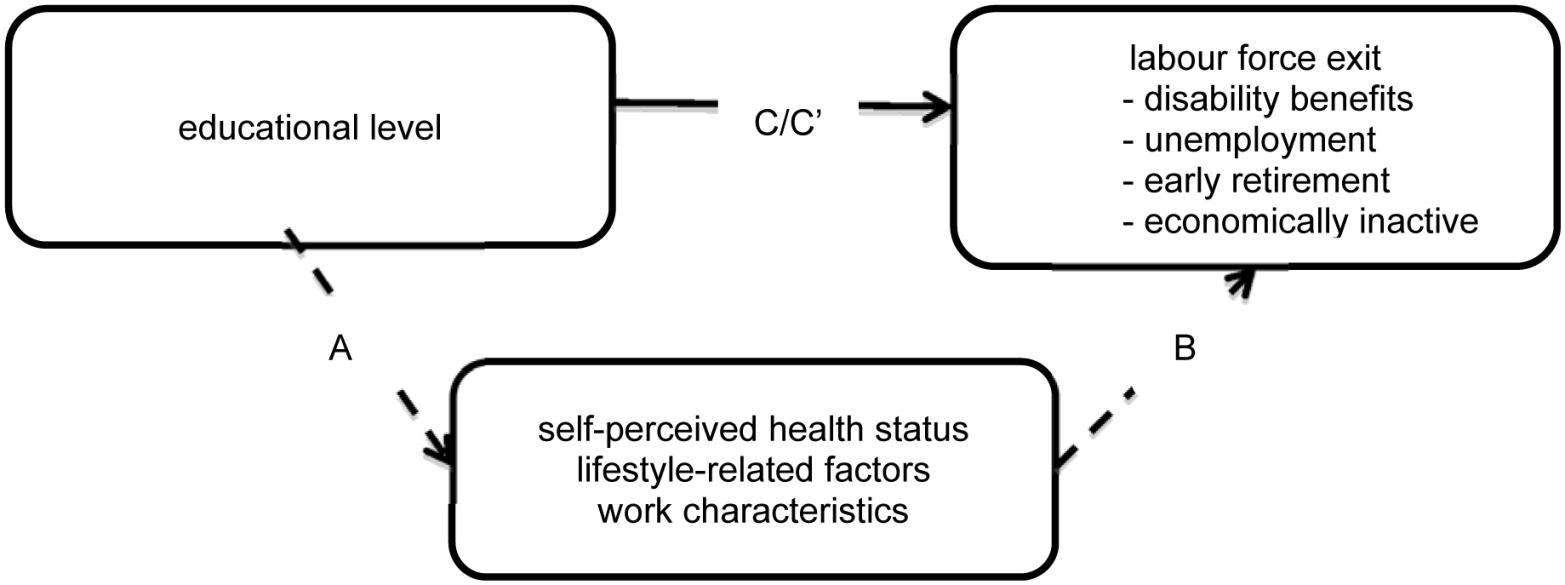
Hypothesized model of the mediating effects of self-perceived health status, lifestyle-related factors and work characteristics on the relation between educational level and exit from paid employment.

The mediating effect of health, lifestyle and work characteristics on the association between education and labour force participation was assessed using a step-approach [25]. The first three steps of the mediation analysis (Step A-C in Fig 1) are described in the previous paragraph. In the final step (step C’), the effect of the mediators on the relation between education and exit from the labour force was assessed by adjusting for the explanatory factors that were statistically significantly associated with both education and at least one of the pathways of exit from paid employment. The percentage change in the β coefficient of educational level after adjustment for the explanatory factors was calculated by: (β base model)- (β adjusted model)/ [(β base model)-1], in which the base model contains education, adjusted for sex, age, and marital status.

All statistical analyses were carried out with STATA version 12.

## Results

The majority of the respondents was male (58.8%) and the mean age was 39.0 years (SD 10.6). Exit from paid employment was most prevalent among workers with a low educational level (Table 1). Becoming economically inactive was the most prevalent pathway of exit from paid employment (25.41 per 1000 person years), followed by unemployment (13.48 per 1000 person years), early retirement (10.24 per 1000 person years), and disability benefits (4.25 per 1000 person years).

**Table 1:** Labour force exit through different pathways stratified by educational level (n = 14708).

	Disability benefits		Unemployment		Early retirement^1^		Economic inactivity	
	n	%	n	%	n	%	n	%
Low education (n = 3509)	138	3.9	396	11.3	287	8.2	635	18.1
Moderate education (n = 7208)	167	2.3	565	7.8	383	5.3	1172	16.3
High education (n = 3991)	83	2.1	270	6.8	265	6.6	514	12.9
Total population(n = 14708)	388	2.6	1231	8.4	935	6.4	2321	15.8

^1^ For early retirement only persons who were 50 years or older were selected (N = 2922).

### Educational inequalities in health, lifestyle and work characteristics

Lower educated workers were more than twice as likely to have a less than good self-perceived health compared with workers having a high education (OR:2.08, 95%CI:1.81–2.37). Table 2 further shows educational inequalities in lifestyle and psychosocial and physical work characteristics, indicating a higher occurrence of an unhealthy lifestyle and–except for job demands–unfavourable work characteristics among lower and intermediate educated workers than higher educated workers.

**Table 2:** Health status, lifestyle-related factors and work characteristics stratified by educational level among employed individuals (n = 14,708) in the Netherlands at baseline.

		High education (n = 3991)		Moderate education (n = 7208)		Low education (n = 3509)	
		%	OR (95%CI)	%	OR (95%CI)	%	OR (95%CI)
health status	poor/moderate	10.3	1.00	11.7	**1.21 (1.06–1.37)**	19.4	**2.08 (1.81–2.37)**
*Lifestyle-related factors*							
smoking		29.3	1.00	40.0	**1.63 (1.50–1.77)**	52.2	**2.73 (2.48–3.01)**
heavy alcohol intake		9.1	1.00	10.2	**1.17 (1.02–1.34)**	12.3	**1.35 (1.17–1.57)**
<1h/week sports		34.5	1.00	46.1	**1.67 (1.54–1.81)**	62.0	**3.08 (2.80–3.38)**
BMI	underweight	1.4	1.00	1.6	1.05 (0.76–1.45)	1.9	**1.89 (1.31–2.72)**
	normal weight	62.0	1.00	57.9	1.00	47.7	1.00
	overweight	31.5	1.00	33.1	**1.26 (1.15–1.37)**	38.3	**1.52 (1.37–1.68)**
	obese	5.1	1.00	7.5	**1.71 (1.44–2.02)**	12.1	**2.97 (2.48–3.55)**
*Work characteristics*							
job demands	high	38.0	1.00	26.7	**0.61 (0.56–0.67)**	22.1	**0.46 (0.42–0.51)**
job control	low	21.4	1.00	32.0	**1.66 (1.51–1.81)**	43.4	**3.06 (2.76–3.39)**
rewards	low	15.6	1.00	18.4	**1.20 (1.08–1.34)**	19.0	**1.33 (1.18–1.50)**
physical job demands	high	7.4	1.00	29.8	**5.31 (4.66–6.04)**	43.2	**9.68 (8.44–11.11)**

Adjusted for sex, age, and marital status, ORs in bold represent statistically significant associations (p<0.05).

### Determinants of exit from paid employment

Lower educated workers had a higher risk of disability benefits (SHR: 1.84, 95%CI: 1.40–2.42), unemployment (SHR: 1.74, 95%CI: 1.49–2.03), and economic inactivity (SHR: 1.53, 95%CI: 1.36–1.71). Educational level was not statistically significantly related with early retirement. Workers with poor or moderate self-perceived health were more likely to exit through disability benefits (SHR: 6.45, 95%CI: 5.26–7.90) and unemployment (SHR: 1.76, 95%CI: 1.53–2.02), but were not at increased risk of early retirement (SHR: 0.97, 95%CI: 0.82–1.14) and economic inactivity (SHR: 0.88, 95%CI: 0.78–1.00).

Smoking, lack of sports participation, and being underweight were related to disability benefits, unemployment and economic inactivity. Those workers reporting an unhealthy lifestyle had no increased risk of early retirement.

Low job control and low rewards, as well as high physical job demands were risk factors for disability benefits. Low job control was also related with unemployment and early retirement. None of the work characteristics was statistically significantly related to early retirement and economic inactivity.

### Mediating effect of health, lifestyle and work characteristics

The mediation analysis is restricted to disability benefits, unemployment and economic inactivity (step C in Fig 1; Table 1), because education was only related with these exit routes. Education was associated with all potential mediators (step A; Table 2). Only heavy alcohol intake and high work demands were not found to be risk factors of the exit pathways (step B; Table 3), and therefore not included in the mediation analysis. Table 4 shows the results of the final step of the mediation analysis (step C’). Self-perceived health status partly mediated the relation between low education and disability benefits (40%) and unemployment (9%), but did not mediate the relation between education and economic inactivity. Lifestyle also partly explained educational differences in exit from paid employment (low education: 14%-31%, moderate education: 14%-54%). Work characteristics were only of influence on the relation between education and disability benefits (low education: 12%, moderate education: 30%). The contribution of specific lifestyle-related factors and work characteristics differed per pathway, as shown in S1 Table. Adjustment for the combination of health, lifestyle and work characteristics attenuated the relation between low educational level and disability benefits with 62% (SHR: 1.26, 95%CI: 0.92–1.74), unemployment with 21% (SHR: 1.56, 95%CI: 1.32–1.84), and economic inactivity with 11% (SHR: 1.46, 95%CI: 1.29–1.66).

**Table 3:** Competing risks analyses on the influence of health, lifestyle-related factors and work characteristics at baseline among employed persons on the likelihood of exit from work during a follow-up period of 10 years (n = 14708).

		Disability benefits (n = 388/14708)	Unemployment (n = 1231/14708)	Early retirement (n = 922/2922)^1^	Economic inactivity (n = 2321/14708)
		SHR (95%CI)	SHR (95%CI)	SHR (95%CI)	SHR (95%CI)
education	high	1	1	1	1
	moderate	1.16 (0.89–1.51)	1.15 (0.99–1.33)	0.94 (0.80–1.10)	**1.20 (1.08–1.33)**
	low	**1.84 (1.40–2.42)**	**1.74 (1.49–2.03)**	0.92 (0.78–1.09)	**1.53 (1.36–1.71)**
health status	poor/moderate	**6.45 (5.26–7.90)**	**1.76 (1.53–2.02)**	0.97 (0.82–1.14)	0.88 (0.78–1.00)
*Lifestyle-related factors*					
smoking		**1.42 (1.16–1.74)**	**1.48 (1.32–1.66)**	**0.77 (0.67–0.89)**	**1.30 (1.20–1.42)**
heavy alcohol intake		0.85 (0.60–1.19)	1.12 (0.94–1.34)	0.99 (0.82–1.20)	1.06 (0.92–1.21)
<1h/week sports		**1.64 (1.34–2.01)**	**1.21 (1.08–1.35)**	0.90 (0.79–1.03)	**1.12 (1.03–1.22)**
BMI	underweight	**2.40 (1.31–4.41)**	1.42 (0.98–2.07)	1.18 (0.51–2.72)	**1.49 (1.16–1.91)**
	normal weight	1.00	1.00	1.00	1.00
	overweight	**1.44 (1.15–1.79)**	0.97 (0.85–1.11)	1.09 (0.95–1.25)	0.92 (0.84–1.02)
	obese	1.22 (0.85–1.74)	1.22 (1.00–1.49)	1.11 (0.90–1.38)	1.01 (0.86–1.18)
*Work characteristics*					
job demands	high	0.91 (0.73–1.14)	**0.84 (0.74–0.95)**	1.06 (0.92–1.22)	0.96 (0.87–1.05)
job control	low	**1.34 (1.08–1.65)**	**1.20 (1.07–1.36)**	1.15 (1.00–1.32)	1.04 (0.95–1.13)
rewards	low	**1.51 (1.19–1.91)**	1.14 (0.99–1.31)	0.90 (0.75–1.08)	1.09 (0.98–1.21)
physical job demands	high	**1.36 (1.10–1.69)**	1.01 (0.89–1.15)	1.01 (0.87–1.17)	1.08 (0.98–1.18)

Adjusted for sex, age, and marital status. SHRs in bold represent statistically significant associations (p<0.05).

^1^ For early retirement only persons who were 50 years or older were selected.

**Table 4:** Mediating effects of self-perceived health status, lifestyle-related factors and work characteristics on the relation between educational level and exit from the labour force among employed persons during a follow-up period of 10 years (n = 14708).

		Disability benefits (n = 388/14708)		Unemployment (n = 1231/14708)		Economic inactivity (n = 2321/14708)	
		SHR (95%CI)	%	SHR (95%CI)	%	SHR (95%CI)	%
Education	Low	**1.84 (1.40–2.42)**		**1.75 (1.50–2.05)**		**1.53 (1.36–1.71)**	
	Moderate	1.16 (0.89–1.51)		**1.16 (1.00–1.34)**		**1.20 (1.08–1.33)**	
	High	1.00		1.00		1.00	
Education + self-perceived health	Low	**1.44 (1.09–1.90)**	-40	**1.66 (1.42–1.94)**	-9	**1.55 (1.38–1.74)**	-3
	Moderate	1.10 (0.84–1.43)	-36	1.14 (0.98–1.31)	-12	**1.20 (1.08–1.33)**	0
	High	1.00		1.00		1.00	
Education + lifestyle-related factors^1^	Low	**1.52 (1.14–2.02)**	-31	**1.56 (1.33–1.84)**	-21	**1.44 (1.27–1.62)**	-14
	Moderate	1.07 (0.82–1.39)	-54	1.10 (0.95–1.27)	-36	**1.17 (1.05–1.30)**	-14
	High	1.00		1.00		1.00	
Education + work characteristics^2^	Low	**1.71 (1.27–2.31)**	-12	**1.77 (1.51–2.08)**	-2	**1.53 (1.35–1.73)**	0
	Moderate	1.11 (0.85–1.46)	-30	**1.17 (1.01–1.36)**	-6	**1.20 (1.08–1.34)**	0
	High	1.00		1.00		1.00	
Education + health + lifestyle + work	Low	1.26 (0.92–1.74)	-62	**1.56 (1.32–1.84)**	-21	**1.46 (1.29–1.66)**	-11
	Moderate	1.02 (0.77–1.34)	-87	1.12 (0.97–1.30)	-24	**1.18 (1.06–1.31)**	-9
	High	1.00		1.00		1.00	

Adjusted for sex, age, marital status. SHRs in bold represent statistically significant associations.

^1^ smoking, lack of sports participation, BMI.

^2^ low job control, low rewards, high physical job demands.

%: percentage change in the log SHRs [β = 100*(β_base model_- β_adjusted model_)/ (β_base model_), where β = ln(SHR)] expressing the relation between educational level and labour force exit after additional adjustment for health, lifestyle-related factors and work characteristics.

## Discussion

Lower educated workers were more likely to exit paid employment through disability benefits, unemployment, and economic inactivity, but not due to early retirement. Furthermore, workers with poor or moderate health, unhealthy lifestyle, and unfavourable work characteristics were more likely to exit paid employment prematurely, particularly through disability benefits and unemployment. Self-perceived health, lifestyle-related factors and to a lesser extent work characteristics contributed to the educational inequalities in disability benefits. Lifestyle-related factors also partly mediated the relation between education and unemployment and economic inactivity.

The finding that lower educated workers were more likely to be displaced from the labour force is in line with other studies which mainly focused on the socioeconomic gradient in disability benefits [10,18,26–28]. The current study showed that a low education is not only a risk factor for disability benefits, but also for unemployment and economic inactivity. The importance of health, lifestyle and work characteristics on leaving the labour force differed per pathway, but particularly influenced the risk of disability benefits.

A meta-analysis showed the importance of health in labour force participation; poor health is a risk factor for disability benefits (RR: 3.61), unemployment (RR: 1.44), and early retirement (RR: 1.27) [9]. Our results corroborate that disability benefits and unemployment are health-driven exit pathways, but the relation between self-perceived health and early retirement was not corroborated. Two possible explanations may be considered. First, early retirement is a voluntary exit route in which other mechanisms may be operating. Particularly financial considerations and social factors are contributing factors to the decision to retire [29,30]. These factors may differ across countries and time periods, depending also on generosity of institutional arrangements with regard to early retirement. Second, the choice for the competing risk approach over the classical survival analysis may have influenced our findings. This can be illustrated by comparing our findings with those from a recent study–using the same data–investigating health inequalities in exit from paid employment [4]. Findings with regard to the influence of health on disability benefits and unemployment were comparable. However, there is a discrepancy regarding the role of health in early retirement. The effect of poor or moderate health using Cox proportional hazards analysis (SHR: 1.22) was not corroborated when using competing risks analysis (SHR: 0.97). These competing risks needs to be taken into account for a reliable prediction regarding the probability that an individual prematurely exits the labour force via a specific pathway at a given time.

Previous studies reported the importance of lifestyle and work characteristics in receiving disability benefits [10,18,26,27,31]. The current study also showed that lifestyle, in particular smoking, a lack of sports participation, and being underweight were risk factors for exiting the labour force, except through early retirement. Unfavourable work characteristics increased the risk of disability benefits, but contributed less to the other exit pathways. Only lack of job control was a risk factor for unemployment. The influence of high job demands differs from the other work characteristics, showing a lower risk of unemployment. This finding is in line with other studies, showing that high job demands are typically reported in higher socioeconomic positions [10,18].

It was hypothesized that health status, lifestyle-related factors, and work characteristics play a role in the mechanisms through which education affects exit from paid employment. For disability benefits, health (40%), lifestyle (31%) and to a lesser extent work characteristics (12%) played a role in educational inequalities. The relation between ill health and labour force participation did not systematically differ between educational groups. Only for disability benefits it was found that among individuals with a high educational level (SHR 8.18, 95%CI: 5.26–12.72) poor health was more strongly related to this exit pathway than among individuals having a low educational level (SHR 4.21, 95%CI: 2.99–5.91). However, although the strength of the relation was stronger among higher educated individuals, poor health was more prevalent among lower educated individuals leading to a higher public health impact in this particular group.

Educational inequalities in unemployment (21%) and economic inactivity (14%) were also most strongly influenced by lifestyle-related factors. In contrast, work characteristics did not play a role in explaining educational inequalities in unemployment and economic inactivity.

These results are in contrast with other studies concluding that physical working conditions are of greater importance in reducing socioeconomic inequalities in disability benefits [18] and sickness absence [32] than lifestyle factors. These differences may be due to different measures of socioeconomic status as well as inclusion of different determinants and outcomes in the analysis. Additional analyses showed that, although lower educated workers were more likely to be exposed to physically demanding work, this factor was associated with disability benefits among higher educated workers (SHR: 2.26, 95%CI: 1.24–4.12), but not among lower educated workers (SHR: 0.78, 95%CI: 0.55–1.11). Other factors (e.g. health) played a more important role in disability benefits among lower educated individuals.

Our results indicate that particularly health promoting interventions are of importance to attenuate educational inequalities in exit from paid employment. Effective interventions aimed at promoting working conditions are also of importance for maintaining a productive workforce, but less likely to decrease educational inequalities. Since interventions may widen as well as reduce socioeconomic inequalities [33,34], attention needs to be paid to whether the interventions are tailored to lower educated workers.

Strengths of this study are the long follow-up period and the use of register data as a source of labour status instead of self-reported labour status, providing reliable information regarding the month of employment transition. Another strength is the use of competing risks regression analyses.

There are some limitations in this study. The study was conducted among a random sample of Dutch workers. Due to the potential influence of welfare regimes on determinants of exit from paid employment, the results cannot directly be generalized to other countries. The annual non-response of the POLS survey was 35–40%, and persons with a low socioeconomic status may be underrepresented. Another limitation concerns the time-varying nature of the studied determinants. Therefore, the influence of changes in determinants on changes in labour force participation could not be investigated. Our analyses were not stratified by sex, since we observed comparable results. Both in male and female workers self-perceived health (M: 38%, F: 43%), lifestyle (M: 23%, F: 43%) and work characteristics (M: 17%, F: 9%) contributed to the educational inequalities in disability benefits, and lifestyle partly mediated the relation between a low educational level and unemployment (M: 11%, F: 28%) and economic inactivity (M: 20%, F: 10%).

Lower educated workers are at increased risk of exit from paid employment. Self-perceived less than good health, unhealthy lifestyle-related factors and unfavourable physical and psychosocial work characteristics are related to early exit from paid employment and partly explain educational inequalities in exit from paid employment, particularly through disability benefits. Health promotion and improving working conditions might be important measures to maintain a productive workforce, particularly among workers with a low education.

## Supporting Information

S1 Table Mediating effects of self-perceived health status, lifestyle-related factors and work characteristics on the relation between educational level and exit from the labour force among employed persons during a follow-up period of 10 years (n = 14708). (DOCX)
