# Supplementary material for: Educational Inequalities in Exit from Paid Employment among Dutch Workers: The Influence of Health, Lifestyle and Work
Source: PLoS One. 2015 Aug 7;10(8):e0134867. doi: 10.1371/journal.pone.0134867 (PMC4529245; doi:10.1371/journal.pone.0134867)
Supplement: S1 Table — (DOCX) [file pone.0134867.s001.docx]

**S1. Table. Mediating effects of self-perceived health status, lifestyle-related factors and work characteristics on the relation between educational level and exit from the labour force among employed persons during a follow-up period of 10 years (n=14708)**

|  | | Disability benefits  (n=388/14708) | | Unemployment  (n=1231/14708) | | Economic inactivity  (n=2321/14708) | |
| --- | --- | --- | --- | --- | --- | --- | --- |
|  |  | SHR (95%CI) | % | SHR (95%CI) | % | SHR (95%CI) | % |
| Education | Low | **1.84 (1.40-2.42)** |  | **1.75 (1.50-2.05)** |  | **1.53 (1.36-1.71)** |  |
|  | Moderate | 1.16 (0.89-1.51) |  | **1.16 (1.00-1.34)** |  | **1.20 (1.08-1.33)** |  |
|  | High | 1.00 |  | 1.00 |  | 1.00 |  |
| Education adj. for smoking | Low | **1.73 (1.31-2.28)** | -10 | **1.61 (1.37-1.88)** | -15 | **1.44 (1.28-1.62)** | -14 |
|  | Moderate | 1.13 (0.87-1.47) | -18 | 1.11 (0.96-1.28) | -30 | **1.17 (1.05-1.30)** | -14 |
|  | High | 1.00 |  | 1.00 |  | 1.00 |  |
| Education adj. for sports | Low | **1.64 (1.24-2.17)** | -19 | **1.69 (1.44-1.98)** | -6 | **1.50 (1.33-1.69)** | -5 |
|  | Moderate | 1.10 (0.85-1.43) | -36 | 1.13 (0.98-1.31) | -18 | **1.19 (1.07-1.32)** | -5 |
|  | High | 1.00 |  | 1.00 |  | 1.00 |  |
| Education adj. for BMI | Low | **1.78 (1.35-2.35)** | -5 | **1.73 (1.48-2.02)** | -2 | **1.53 (1.36-1.72)** | 0 |
|  | Moderate | 1.15 (0.88-1.49) | -6 | 1.15 (0.99-1.33) | -6 | **1.20 (1.09-1.34)** | 0 |
|  | High | 1.00 |  | 1.00 |  | 1.00 |  |
| Education adj. for job control | Low | **1.76 (1.33-2.33)** | -7 | **1.70 (1.45-1.98)** | -5 | **1.53 (1.36-1.72)** | 0 |
|  | Moderate | 1.14 (0.88-1.49) | -12 | 1.14 (0.98-1.32) | -12 | **1.20 (1.08-1.33)** | 0 |
|  | High | 1.00 |  | 1.00 |  | 1.00 |  |
| Education adj. for rewards | Low | **1.83 (1.39-2.42)** | -1 | **1.74 (1.49-2.03)** | -1 | **1.51 (1.35-1.70)** | -3 |
|  | Moderate | 1.15 (0.88-1.50) | -6 | 1.15 (1.00-1.33) | -6 | **1.19 (1.08-1.33)** | -5 |
|  | High | 1.00 |  | 1.00 |  | 1.00 |  |
| Education adj. for physical job demands | Low | **1.72 (1.28-2.32)** | -11 | **1.81 (1.54-2.12)** | 6 | **1.53 (1.36-1.73)** | 0 |
|  | Moderate | 1.12 (0.85-1.46) | -24 | **1.18 (1.01-1.36)** | 12 | **1.20 (1.08-1.34)** | 0 |
|  | High | 1.00 |  | 1.00 |  | 1.00 |  |

Adjusted for demographic confounders: age, sex, marital status. HRs in bold represent statistically significant associations.

SHR: subdistrbution hazard ratios, 95%CI: 95% confidence interval.

%: percentage change in the log SHRs [β= 100*(β_base model_- β_adjusted model_)/ (β _base model_), where β=ln(SHR)] expressing the relation between educational level and labour force exit after additional adjustment for health, lifestyle-related factors and work characteristics.
